# Lower genetic variability of HIV-1 and antiretroviral drug resistance in pregnant women from the state of Pará, Brazil

**DOI:** 10.1186/s12879-017-2392-y

**Published:** 2017-04-12

**Authors:** Luiz Fernando Almeida Machado, Iran Barros Costa, Maria Nazaré Folha, Anderson Levy Bessa da Luz, Antonio Carlos Rosário Vallinoto, Ricardo Ishak, Marluisa Oliveira Guimarães Ishak

**Affiliations:** 1 Virology Laboratory, Institute of Biological Sciences, Federal University of Pará, Augusto Correa 1, Guama, Belem, Para CEP 66075-110 Brazil; 2 Reference Unit Specialized in Maternal-Child and Adolescent Care, Alcindo Cacela 1421, Sao Bras, CEP 66040-020, Belem, Pará Brazil

**Keywords:** HIV-1, Pregnant, Molecular epidemiology

## Abstract

**Background:**

The present study aimed to describe the genetic diversity of HIV-1, as well as the resistance profile of the viruses identified in HIV-1 infected pregnant women under antiretroviral therapy in the state of Pará, Northern Brazil.

**Methods:**

Blood samples were collected from 45 HIV-1 infected pregnant to determine the virus subtypes according to the HIV-1 *protease* (PR) gene and part of the HIV-1 *reverse transcriptase* (RT) gene by sequencing the nucleotides of these regions. Drug resistance mutations and susceptibility to antiretroviral drugs were analyzed by the Stanford HIV Drug Resistance Database.

**Results:**

Out of 45 samples, only 34 could be amplified for PR and 30 for RT. Regarding the PR gene, subtypes B (97.1%) and C (2.9%) were identified; for the RT gene, subtypes B (90.0%), F (6.7%), and C (3.3%) were detected. Resistance to protease inhibitors (PI) was identified in 5.8% of the pregnant, and mutations conferring resistance to nucleoside reverse transcriptase inhibitors were found in 3.3%, while mutations conferring resistance to non-nucleoside reverse transcriptase inhibitors were found in 3.3%.

**Conclusions:**

These results showed a low frequency of strains resistant to antiretroviral drugs, the prevalence of subtypes B and F, and the persistent low transmission of subtype C in pregnant of the state of Pará, Brazil.

## Background

The extensive genetic diversity of HIV-1 is one of its main features and allows the classification of the strains isolated in various parts of the world into four groups (M, N, O, and P). The M group may be further divided into subtypes, subsubtypes, circulating recombinant forms (CRF), and unique recombinant forms (URF) [1]. The comprehensive description of the genetic diversity of HIV-1 in different geographic regions in Brazil has a great impact on the epidemiological surveillance and may influence the diagnosis as well as the development of vaccines.

In Brazil, 798,366 cases of AIDS were reported through June 2015 [2], which corresponds to one third of cases recorded in South and Central America. The North region of Brazil comprises 5.7% of the reported cases and has an AIDS incidence rate of 25,7/100,000 people, with the largest number of cases (43.47%) occurring in Pará [2].

In the period between 2000 and 2015, 92,210 cases of HIV-1 were reported among pregnant women in Brazil; of which, 6548 cases (7.1%) occurred in the North region and the highest number was observed in the state of Pará (39.92%), followed by the states of Amazonas (3.08%) and Rondônia (6.98%) [2].

The majority of the studies reporting the genetic diversity of HIV-1 in Brazil are associated with the South and Southeast regions of the country as these regions were the epicenter of the HIV/AIDS epidemic in the 1980s. The South region of Brazil presents a different pattern compared to other regions in the country, with a high prevalence of subtype C and several recombinant forms (BC, BF1, and CRF31_BC) [3]. In the South [4] and Northeast [5] regions, subtypes B and F1 are the most prevalent, although various recombinant forms have been found.

The North region of Brazil, the largest land area of the country, circulating HIV-1 subtypes have been described in the states of Amazonas (B and F) [6], Amapá (B and F), and Pará and have presented higher HIV-1 genetic diversity (B, F, C, D, and the recombinant form CRF02_AG). These data demonstrate the importance of Pará as a port of HIV entry in Northern Brazil [7]. The present study describes the circulating HIV-1 subtypes among HIV-1-infected pregnant women as well as the resistance profile of the strains isolated in the state of Pará, Northern Brazil.

## Methods

### Regions and population studied

The study participants were women from the Reference Unit for Special Infectious and Parasitic Diseases (URE-DIPE) and the Reference Unit for Maternal-Child and Adolescent care (UREMIA) in Belém, state of Pará, Brazil.

This study included the entire population of HIV-1-infected pregnant women (*n* = 45) who received care from April 2007 to October 2008. These women were referred to the URE-DIPE and UREMIA by the Health Care Units located in 14 cities: Belém, (18) São Miguel do Guamá, (1) Paragominas, (1) Moju, (1) Concórdia do Pará, (1) Abaetetuba, (2) Marabá, (1) Marituba, (3) Mãe do Rio, (1) Igarapé-Miri, (1) Icoaraci, (3) Castanhal, (2) Canaã dos Carajás, (1) and Ananindeua (9).

This study was approved by the Human Research Ethics Committee of the João de Barros Barreto University Hospital, protocol number 2090/05. All patients signed a free and informed consent form at the time of sample collection and answered a structured epidemiological questionnaire (through an interview) applied by either a physician of the Unit that performed the clinical monitoring or a member of the study. The questionnaire included demographic and epidemiological information such as the date of the first positive HIV-1 test, history of partners from other states or countries, use of the antiretroviral therapy (ART), date when the patients started ART, and the stage of pregnancy when HIV-1 was diagnosed (for those women who did not know they were HIV-1 positive). Moreover, other information was obtained from the participants’ medical records such as the T CD4^+^ lymphocyte (CD4TL) count and plasma HIV-1 viral load; information was obtained from the reference units protocols, using data from the most recent tests at the time of interview.

Blood samples (5 mL) were collected in two tubes containing EDTA using a vacuum system. The samples (plasma and cellular components) were transported to the Virology Laboratory at the Institute of Biological Sciences, Federal University of Pará, and stored at −70 °C until use.

### Polymerase chain reaction (PCR)

DNA was extracted from the cellular components of the blood using a Puregene kit (Puregene, Gentra Systems, Inc., USA) according to the manufacturer’s instructions. The nucleic acid was eluted and stored at −20 °C until use. Subsequently, DNA was submitted to a nested PCR to amplify part of the region of the HIV-1 RT and HIV-1 PR. The first PCR cycle was performed using 4 μg of extracted DNA, 125 mM of each dNTP (Perkin-Elmer, USA), 20 pmol/μL of each of the two external primers, 2.5 mM MgCl_2_ and 10× buffer (Perkin-Elmer, USA) in a final volume of 50 μL. The reactions were performed in a thermocycler (Perkin-Elmer, USA) for 5 min at 94 °C followed by 35 cycles at 94 °C (40 s), 50 °C (40 s), and 72 °C (1 min) and a 10 min extension at 72 °C. Five microliters of the amplified product was used in the nested PCR along with a set of internal primers in a final volume of 50 μL. The same incubation times and temperatures of the first reaction were used. For the PR, DP10/DP11 were used as external primers, and DP16/DP17 were used as internal primers [8]. For amplification of the fragment of the RT gene, RT09/RT12 were used as external primers, and RT01/RT04 were used as internal primers [9]. The amplified products were analyzed by electrophoresis on a 2% agarose gel. After amplification, the fragments were purified using the QIA Quick Purification Kit (Qiagen, USA) prior to nucleotide sequencing.

### HIV-1 genetic subtypes, nucleotide sequencing and phylogenetic analysis

A 2 μL aliquot of PCR product was sequenced using the Big Dye Terminator Kit (Applied Biosystems, Foster City, CA, USA) on an automatic sequencer (DNA sequencer model 310, Biosystems, Foster City, CA, USA).

Prior to the phylogenetic analysis, an HIV-1 gene subtyping tool available on the website (https://hivdb.stanford.edu/hivdb/by-mutations/) was used to determine whether the obtained sequences were pure subtypes or CRFs.

The nucleotide and amino acid alignments were performed using the BioEdit version 5.0.9 software program [10]. The evolutionary distance was estimated using Kimura’s two parameter model, and the phylogenetic trees were constructed using the neighbor-joining and maximum likelihood methods; the reliability of the tree topology was tested by bootstrap analysis with 1000 replicates using the MEGA 5.0 software program [11]. The sequences determined in this study were aligned with the reference sequences of the subtypes obtained from the Los Alamos database (https://www.hiv.lanl.gov/content/sequence/HIV/mainpage.html), which are representative of the current HIV-1 strains circulating around the world.

### Resistance analysis

Drug resistance mutations (DRM) and susceptibility to antiretroviral drugs were analyzed using the Calibrated Population Resistance (CPR) tool (version 5.0 beta, available at http://cpr.stanford.edu/cpr.cgi). The antiretroviral susceptibility mutation profiles were analyzed using the Stanford HIV Drug Resistance Database (hivdb.stanford.edu).

## Results

Blood samples were collected from 45 HIV-1-infected pregnant women receiving ART in the city of Belém, Pará, Brazil, from April 2007 to October 2008. The mean age was 25.06 years (ranging from 14 to 37 years), and the majority of the women (64.4%) were three to six months pregnant. Table 1 shows the main demographic characteristics of the group. Most women reported being single (64.4%), having only basic education (48.9%), without an occupation other than housewives (60%).

**Table 1:** Demographic and social characteristics of HIV-1-infected pregnant women from the state of Pará, Brazil

Socio-demographic characteristics		N
Age range	14–23	13
	24–33	27
	34–37	5
Marital status	Married	14
	Single	29
	Separated/Divorced	2
Educational level	Primary school	22
	High School	20
	College education	3
Occupation	Household	27
	Student	5
	Others	13

Regarding the behavioral aspects analyzed, 51.1% mentioned having a heterosexual partner, 20% assumed having relationships with HIV-1 positive partners, 24.4% had a partner who was a non-injecting drug user, and 60% denied having had anal intercourse. The vast majority of women (75.6%) reported having only one partner per month, 62.2% had a relationship with people from other states of Brazil, and 20% had already been diagnosed with a sexually transmitted infection (STI). Regarding ART, 29 women (64.4%) were using therapy at the time of sample collection, with time ranging between three months to four years, 12 women (26.7%) had not yet started therapy, and 3 women (6.7%) reported having stopped the treatment against HIV at least once.

Among the pregnant women, 31 (68.9%) had other children (mean 2.54 children), while 14 (31.1%) were experiencing their first pregnancy. It is noteworthy that 22 women who already had children (48.9%) reported that they did not attend a prenatal care for any of the pregnancies, and therefore, they breastfed their first children and stopped after the diagnosis of HIV infection. Only two children (4.4%) were found to be infected by HIV-1.

The values of CD4TL count were obtained from 28 participants, and plasma HIV-1 viral load was obtained from 23 participants (Table 2). There was no statistically significant association between the stage of pregnancy, use of ART, mean CD4TL count (365.04 ± 236.25 cells/μL, ranging from 122 to 1210 cells/μL), and mean plasma HIV-1 viral load (log_10_ 3.699, ranging from 2.699 to 4.699).

**Table 2:** Characteristics of HIV-1-infected pregnant women from the state of Pará, Brazil in relation to subtypes of HIV, CD4TL numbers, viral load, mutations of PR and RT and use of antiretroviral therapy

ID	Subtypes PR/RT	CD4TL	VL (Log_10_)	Mutations PR	Mutations RT	ARV
14,012	B/−	234	4.079			No
14,012	B/−	-^§^	-^§^			Yes
14,017	B/B	301	4.041			No
14,045	B/B	-^§^	-^§^	M46I, G48E, L10I, A71V		Yes
14,078	B/B	-^§^	-^§^			Yes
14,319	B/B	611	2.929			Yes
14,566	B/B	-^§^	-^§^			Yes
14,599	B/B	275	4.061			No
14,644	−/B	231	3.903			Yes
14,645	−/B	-^§^	-^§^			Yes
14,648	−/B	288	4363			Yes
14,749	B/−	317	3.041			Yes
14,993	B/B	-^§^	-^§^			Yes
15,275	B/B	122	4.699		K70E	Yes
15,276	−/B	-^§^	-^§^			Yes
15,277	C/C	451	3.740			Yes
15,278	B/B	389	3.875	I84V		Yes
15,364	B/B	-^§^	-^§^			Yes
15,365	B/−	818	2.813			Yes
15,366	B/−	312	-^§^			Yes
15,367	B/−	-^§^	-^§^			Yes
15,395	B/B	416	3.845			Yes
15,665	B/B	-^§^	-^§^			Yes
16,071	B/B	276	3.114			No
16,072	B/B	232	4.322			No
16,077	B/B	-^§^	-^§^			Yes
16,078	B/B	1210	2.699		K103 N	Yes
16,214	−/F	352	3.968			Yes
16,557	−/B	-^§^	-^§^			Yes
16,558	−/B	187	4.146			No
16,689	B/B	417	3.698			No
16,789	−/B	375	-^§^			Yes
17,037	B/−	474	3.602			No
17,176	B/−	246	-^§^			Yes
17,177	B/−	-^§^	-^§^			Yes
17,182	B/−	-^§^	-^§^			Yes
17,185	−/B	265	-^§^			Yes
17,191	B/−	359	2.954			Yes
17,196	B/B	311	3.978			Yes
17,200	−/B	220				Yes
17,227	−/F	129	4.025			No
17,229	B/−	403	3.176			No
17,258	B/−	-^§^	-^§^			Yes
17,569	B/−	-^§^	-^§^			No
17,591	B/−	-^§^	-^§^			Yes
17,796	B/−	-^§^	-^§^			No

(−) not amplified; (−^§^) not informed

**Fig. 1:**
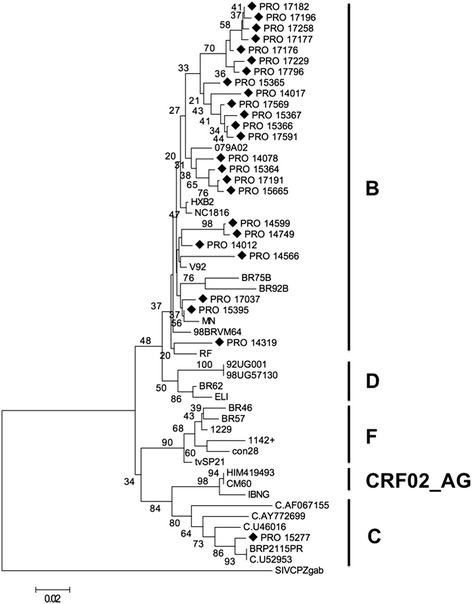
Phylogenetic analysis based on the alignment of 249 nucleotides of the *pro* gene of samples from Pará (♦) with reference strains from the *Los Alamos* database. The sequence SIV_CPZgab_ was used as an outgroup. The tree was constructed using the Kimura 2-parameter neighbor-joining (NJ) method. The numbers on the nodes of the tree indicate the bootstrap value obtained using 2000 replicates. The scale bar represents a difference of 2%

**Fig. 2:**
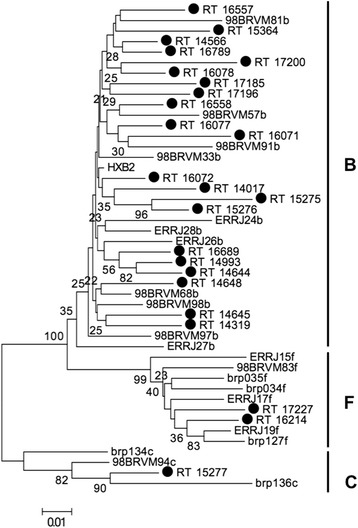
Phylogenetic tree based on the alignment of the 450 nucleotides of the *reverse transcriptase* gene of 24 samples from Pará (•) with reference strains from the *Los Alamos* database. The tree was constructed using the Kimura 2-parameter neighbor-joining (NJ) method. The numbers on the nodes of the tree indicate the bootstrap value obtained using 2000 replicates. The scale bar represents a difference of 1%

The PR gene region was successfully amplified in 34 samples, and the phylogenetic tree showed that 97.1% of the sam ples were subtype B and 2.9% were subtype C (Fig. 1). However, the RT region (Fig 2) was amplified in 30 samples (which were not necessarily the same samples amplified for the PR gene region), and showed that 90.0% were classified as subtype B, 6.7% as subtype F, and 3.3% as subtype C. Only 19 samples were amplified for both regions and no recombinant forms was observed. The information of CD4TL, viral load, PR mutation, RT mutations and ART treatment status as shown in Table 2.

Four women presented HIV-1 strains with some sign of resistance to ARV, being two (5.8%; #14045 and #15278) to protease inhibitors (PIs), one (3.3%; #15275) to nucleoside reverse transcriptase inhibitors (NRTIs) (#15275), and one (3.3%; #16078) to non-nucleoside reverse transcriptase inhibitors (NNRTIs).

Woman #14045 (infected with HIV-1; subtype B^PR^/B^RT^; IP mutations M46I, G48E, L10I, A71V) was 26 years old, lived in Belém, had been using ART since 2005, had three children who were not infected with HIV-1, attended prenatal care during all pregnancies, but she reported stopping her treatment at some point. Another woman (#15278) (HIV-1; subtype B^PR^/B^RT^; IP mutation I84V) lived in Abaetetuba, which is a town close to Belém, and had been using ART since 2006, without interruption. She was 27 years old and had two children who were not infected with HIV-1; she and her partner were non-injecting drug users.

Woman #15275 (HIV-1, subtype B^PR^/B^RT^; NRTI mutation K70E) was 29 years old, lived in Belém, had two children, did not breastfeed, had only one partner, who was also HIV-positive, had been using ART since 2006, and reported previous interruption of treatment. Pregnant woman #16078 (HIV-1; subtype B^PR^/B^RT^; NNRTI mutation K103 N) was 19 years old, was living in Ananindeua, had only one child, did not breastfeed, was a housewife and single, had only one partner who was HIV positive, had been using ART since 2006, and reported previous interruption once.

## Discussion

The lack of information associated with low educational level are risk factors for HIV-1 exposure in Brazil [3], and the present study was consistent with the findings that are usually observed: infected persons are young women with low educational level, which currently reflects the population more affected by HIV-1 infection in Brazil [2].

Other social and behavioral information obtained from the examined women also show variables that are often associated with higher risk of transmission of HIV-1 and other STIs. The consistent use of condoms during intercourse was low, and 20% of the surveyed women had contact with HIV-1-positive individuals. Although drugs were not used intravenously, this variable promotes other variables of risk, including unprotected sexual relations with anal intercourse, as previously shown in other studies [12, 13]. Even with the existence of a free treatment program widely distributed across the country, a large percentage of this population was not using ART, and even worse, nearly one third of this population had never used the treatment facilities. Difficulties with ART adherence by HIV-1-positive individuals are often reported, and this could be one explanation for the large number of women who do not receive treatment [14].

Although the immunological response is physiologically compromised during pregnancy and a decrease in the CD4TL count is usually observed [15], no significant difference was found between the mean value of these cells and the pregnancy stage in the present study, which has also been observed in other studies [16, 17].

Studies on the molecular epidemiology of HIV-1 in Brazil show that the B subtype is the most prevalent strain in all described geographical regions of the country, in addition to the decreasing prevalence of the C, D, and F subtypes and the recombinant forms [5, 18–21]. In the present study, the B subtype was also the most prevalent, followed by the F and C subtypes, which had been previously described in the cities of Belém and Macapá in the adult population [8]. It is important to mention that the F subtype could only be found when the RT gene was amplified, which demonstrates the need to amplify at least two genes in studies addressing the occurrence of subtypes and recombinant forms of the virus [22].

The prevalence of subtypes B and F demonstrated in the present study has also been observed in other areas of South America [23, 24]. Most of the women had only one partner, although 20% of them reported relationships with HIV-1-positive men. It is possible that this low activity for HIV-1 transmission did not facilitate the occurrence of recombinant forms of the virus. Moreover, the majority of the patients were already using ART.

Two pregnant women, one living in Belém (#14045) and another living in Abaetetuba (#15278), were carriers of strains with mutations in the protease region (M46I, G48E, L10I, A71V, and I84V). The low prevalence of HIV-1 strains that are resistant to this class of drugs reflects the low circulation of viruses with resistance to PIs in the North region of the country [25], differently from what was observed in Rio de Janeiro [26] and in Goias [27] with a prevalence level around 16%.

The most frequently occurring mutation that conferred resistance to PIs was the M46I mutation, which can be a good indicator of regional differences in HIV-1 strains circulating in the country. However, eight or more mutations are required for a strain to be considered resistant, while partial resistance can be observed by the accumulation of five to seven of these mutations in addition to the combination of several primary and secondary mutations that do not always confer resistance [28]. This resistance development process is complex and requires the accumulation of mutations, which was not observed in the present study. Three mutations that confer resistance to atazanavir were identified in sample #14045 (G16E, M46I, and I62V) and two in sample #15278 (I62V and I84V).

The NRTIs initially used as ART included regimens consisting of tenofovir/emtricitabine, zidovudine/lamivudine, and abacavir/lamivudine with a combination of doses [29]. The M41 L and D67N mutations confer resistance to zidovudine and stavudine [28, 30]. The M184 V mutation confers resistance to lamivudine as well as to abacavir and emtricitabine [28, 31]. The K70E mutation (#15275), which is associated with resistance to NRTIs, and the K103 N mutation (#16078), which is associated with resistance to NNRTIs, were observed in two strains. None of these mutations confers a degree of resistance that is noteworthy for the two classes of drugs used in ART.

## Conclusions

The present study indicated that in Pará, Northern Brazil, the frequency of circulating ART-resistant HIV-1 strains is still low among pregnant women, which may suggest the success of the therapy currently used in the country, shown by the absence of mutations indicative of primary resistance in the investigated group. Although the study was performed with samples collected in 2007 and 2008, this is the first approach to study HIV-1 among pregnant women in the state of Para and it will certainly serve as a basis for future studies. The prevalence of subtype B demonstrated that these groups of pregnant women present the same epidemiological pattern as the rest of the population and, in fact, present a lower frequency of other subtypes observed in the region.

## Abbreviations

ART: Antiretroviral therapy
CD4TL: T CD4^+^ lymphocyte
CRF: Circulating recombinant forms
IDU: Injectable drug user
NIDU: Non-injectable drug user
NNRTI: Non-nucleoside reverse transcriptase inhibitor
NRTI: Nucleoside reverse transcriptase inhibitor
PI: Protease inhibitor
RT: Reverse transcriptase
STI: Sexually transmitted infection
URE-DIPE: Reference Unit for Special Infectious and Parasitic Diseases
UREMIA: Reference Unit for Maternal-Child and Adolescent care
URF: Unique recombinant forms
